# Photopheresis Abates the Anti-HLA Antibody Titer and Renal Failure Progression in Chronic Antibody-Mediated Rejection

**DOI:** 10.3390/biology10060547

**Published:** 2021-06-18

**Authors:** Marilena Gregorini, Claudia Del Fante, Eleonora Francesca Pattonieri, Maria Antonietta Avanzini, Maria Antonietta Grignano, Irene Cassaniti, Fausto Baldanti, Giuditta Comolli, Angela Nocco, Miriam Ramondetta, Gianluca Viarengo, Vincenzo Sepe, Carmelo Libetta, Catherine Klersy, Cesare Perotti, Teresa Rampino

**Affiliations:** 1 Department of Internal Medicine and Therapeutics, University of Pavia, 27100 Pavia, Italy; carmelo.libetta@unipv.it; 2 Unit of Nephrology, Dialysis and Transplantation, Fondazione IRCCS Policlinico San Matteo, 27100 Pavia, Italy; ef.pattonieri@gmail.com (E.F.P.); wta87@hotmail.it (M.A.G.); v.sepe@smatteo.pv.it (V.S.); t.rampino@smatteo.pv.it (T.R.); 3 Immunohematology and Transfusion Service, IRCCS Policlinico San Matteo, 27100 Pavia, Italy; c.delfante@smatteo.pv.it (C.D.F.); gl.viarengo@smatteo.pv.it (G.V.); c.perotti@smatteo.pv.it (C.P.); 4 Immunology and Transplantation Laboratory, Cell Factory, Pediatric Hematology Oncology, Fondazione IRCCS Policlinico S. Matteo, 27100 Pavia, Italy; ma.avanzini@smatteo.pv.it; 5 Molecular Virology Unit, Department of Microbiology and Virology, Fondazione IRCCS Policlinico San Matteo, 27100 Pavia, Italy; i.cassaniti@smatteo.pv.it (I.C.); f.baldanti@smatteo.pv.it (F.B.); g.comolli@smatteo.pv.it (G.C.); 6 Department of Clinical, Surgical, Diagnostic and Pediatric Sciences, University of Pavia, 27100 Pavia, Italy; 7 Experimental Research Laboratories, Biotechnology Area, Fondazione IRCCS Policlinico S. Matteo, 27100 Pavia, Italy; 8 Laboratory of Transplant Immunology, Fondazione IRCCS Ca’ Granda, Ospedale Maggiore Policlinico Milano, 20122 Milano, Italy; angela.nocco@policlinico.mi.it (A.N.); miriam.ramondetta@policlinico.mi.it (M.R.); 9 Clinical Epidemiology and Biometry Unit, Fondazione IRCCS Policlinico San Matteo, 27100 Pavia, Italy; c.klersy@smatteo.pv.it

**Keywords:** lymphocytes subset, chronic allograft rejection, kidney transplantation, extracorporeal photopheresis, proteinuria, Donor-Specific-Antibody

## Abstract

**Simple Summary:**

The most common cause of late allograft failure is chronic active antibody-mediated rejection (ABMR), but no effective therapy is available. Different immunosuppressive drugs in combination with procedures that remove serum antibodies have been used and the results have not shown to improve graft and patient outcome, but only an increased risk of adverse events. Extracorporeal pho-topheresis (ECP) is leukapheresis-based immunomodulatory therapy not associated with adverse effect, in which lymphocytes treat-ed with 8-methoxypsoralen (8-MOP) are irradiated with ultraviolet-A (UVA) ex vivo and re-infused into the patient. In this study we investigated therapeutic long-term effect of ECP in patients with biopsy proved chronic ABMR.

**Abstract:**

Objective: Chronic renal antibody-mediated rejection (ABMR) is a common cause of allograft failure, but an effective therapy is not available. Extracorporeal photopheresis (ECP) has been proven successful in chronic lung and heart rejection, and graft versus host disease. The aim of this study was to evaluate the effectiveness of ECP in chronic ABMR patients. Patients and Methods: We investigated ECP treatment in 14 patients with biopsy-proven chronic ABMR and stage 2–3 chronic renal failure. The primary aim was to e valuate the eGFR lowering after 1 year of ECP therapy. The ECP responders (R) showed eGFR reduction greater than 20% vs the basal levels. We also evaluated the effectiveness of ECP on proteinuria, anti-HLA antibodies (HLAab), interleukin 6 (IL-6) serum levels, and CD3, CD4, CD8, CD19, NK, Treg and T helper 17 (Th17) circulating cells. Results: Three patients dropped out of the study. The R patients were eight (72.7%) out of the 11 remaining patients. Because ECP was not associated with any adverse reaction, the R patients continued such treatment for up to 3 years, showing a persisting eGFR stabilization. Twenty four hour proteinuria did not increase in the R patients over the follow-up when compared to the non-responder patients (NR). In the R patients, the HLAab levels were reduced and completely cleared in six out of eight patients when compared with the NR patients. The NR HLAab levels also increased after the discontinuation of the ECP. The ECP in the R patients showed a decrease in CD3, CD4, CD8, CD19, and NK circulating cells. The ECP treatment in the R patients also induced Tregs and Th17 cell increases, and a decrease of the IL-6 serum levels. Conclusions: ECP abates the HLAab titer and renal failure progression in patients with chronic renal ABMR, modulating the immune cellular and humoral responses.

## 1. Introduction

The development of new immunosuppressive drugs has improved graft outcomes, particularly by defeating acute rejection, the main cause of graft failure in the early post-transplant period. In contrast, it is disappointing that saving the graft from early failure has not improved long term survival. Most grafts undergo a slow and steady fibrogenic process, eventually leading to organ failure [1].

This phenomenon has been named Chronic Transplant Nephropathy (CTN) in Banff’s classification, and reflects the variety of fibrogenic effectors and the difficult distinction of their specific role [2,3].

CTN shows tubular atrophy, interstitial fibrosis and glomerular/vascular sclerosis that are the final steps of scarring, whatever the cause. Further investigations disclosed that what seemed to be a uniform wasted land displayed tissue feature expressions of specific pathogenic effectors. A major understanding was the recognition of tissue indicators, like C4d deposits, that identify a clinically silent, slowly progressing chronic rejection that is caused by antibodies [4,5]. Although the cause of the injury mediated by donor-specific alloantibodies (DSA) against human leukocyte antigen (HLA) and non-HLA antigens has not been fully elucidated, several potential mechanisms have been proposed, which include: (1) direct DSA-mediated injury to the endothelium, (2) indirect injury via complement activation, and (3) the DSA-induced recruitment of inflammatory cells [6].

Different immunosuppressive drugs (intravenous immunoglobulin (IVIg) and/or rituximab, bortezomib or eculizumab) and/in combination with procedures to remove serum antibodies (plasmapheresis or immunoadsorption) have been used to reduce DSA production. However, the effectiveness of the graft function and patient outcomes are still under debate [7,8,9,10,11,12,13,14,15,16,17,18,19,20,21]. In addition, Mesenchymal Stem Cell therapy, known for its anti-inflammatory and immunomodulatory effects [22,23,24] has been investigated in chronic antibody-mediated rejection (ABMR), but with variable results [25,26]. A few pieces of recent evidences have shown that tocilizumab in ABMR patients stabilizes graft function by decreasing DSA levels, although bacterial and viral infections have been reported during the follow-up [27,28]. However, most of the trials were observational studies and were not randomized, controlled trials with adequate statistical power to compare the safety and efficacy of different therapeutic strategies. The recommended treatment based on the available evidence and expert consensus from the Transplantation Society Working Group is the optimization of immunosuppression and supportive care, with the reintroduction of steroids (if on a steroid-free regimen), maintaining the trough tacrolimus levels at >5 ng/mL, and medical management optimization [8].

It was argued that prevention rather than late therapy could save the graft, but protocol biopsies revealed that chronic rejection starts earlier than expected, often while full immunosuppression is still delivered; therefore, it would seem unproductive to prolong and/or strengthen the pharmacologic intervention.

Extracorporeal photopheresis (ECP) is a procedure originally developed to treat cutaneous lymphoma. Briefly, it consists of the induction of the apoptosis of mononuclear cells (MNCs) loaded in vitro with psoralen (8-MOP), a photosensitizing agent, then irradiated with Ultraviolet A. The reinjection of apoptotic MNCs causes a tolerogenic modulation of the T cells, with a shift from Th1 to Th2 subpopulations and a rise of circulating Tregs. The exact mechanisms by which inducing apoptosis with ECP deviates T cells to tolerogenic phenotypes are not clear, nor it is known whether ECP targets other cells, such as T lymphocytes and dendritic cells. However, ECP has been used to treat graft versus host disease (GVHD) and heart and lung transplant rejection, and the results are encouraging [29,30,31,32,33].

The retrospective collection of the effects of ECP in renal transplants indicates that the procedure can save the renal function in patients with acute antibody-driven rejection, but only anecdotal data are available about the effects of ECP on chronic ABMR [34,35,36].

Recently, a study carried out in our hospital gave evidence that ECP improves the organ function and death risk in the recipients of lung transplants with chronic rejection [37], an impressive success that has been the thrust to start the present prospective investigation aiming to evaluate the effect of ECP in chronic ABMR.

The results obtained to date in a limited but significant number of patients seem to be very encouraging and worthy to be made available to the renal transplant community as the basis for further confirmatory studies.

## 2. Methods

### 2.1. Study Design and Patients

A prospective observational study was performed on 14 patients with a diagnosis of chronic ABMR.

The study protocol was approved by the Ethics Committee (E-20170031747) and was in full compliance with the requirements of the Helsinki Declaration of 2000 [38].

The primary aim was to evaluate the effect of ECP on the estimated glomerular filtration rate (eGFR) decline.

The non-responder patients (NR) were defined as the patients in which eGFR decreased more than 20% with respect to the baseline value after 12 months from start of the ECP treatment.

The responder patients (R) were defined as the patients in which eGFR decreased less than 20% with respect to the baseline value after 12 months from the start of ECP.

The secondary aims were the evaluation of the effect of ECP on: proteinuria (PTU)/24 h, donor and non-donor specific circulating anti-HLA antibody levels, CD3, CD4, CD8, CD19, NK cells, Tregs cells, T helper 17 cells (Th17), and interleukin 6 (IL-6) serum levels.

Blood and urine samples were collected before the ECP treatment, at 1 year and every year throughout the follow up.

The inclusion criteria needed for enrolment were: signed informed consent, renal biopsy-proven chronic antibody-mediated kidney rejection (Banff 2013), eGFR > 20 mL/min/1.73sqm, 24-h proteinuria > 0.5 g/, ACE inhibitor and/or Angiotensin2 receptor antagonist therapy if the patient had hypertension, calcineurin inhibitor drugs, no therapy with rituximab/bortezomib/eculizumab in the last 12 months, no cancers and no pregnancy.

Chronic ABMR diagnosis required the presence of histologic lesions of chronic tissue injury at biopsy and at least one of the following criteria: (a) evidence of current/recent antibody interaction with the vascular endothelium (linear C4d staining in peritubular capillaries), and (b) the presence of circulating donor specific antibodies (DSA). [39]

In particular, all of the enrolled patients showed, at biopsy, the evidence of chronic transplant glomerulopathy and plurifocal areas of tubular atrophy/interstitial fibrosis.

### 2.2. Anti-HLA Antibodies

The presence of anti-HLA antibodies (DSA and not-DSA) was tested using Luminex technology. Serum samples from the recipients were analyzed for class I and class II IgG HLA antibodies using the commercially available LABScreen Single Antigen Beads Class I and Class II Assay Kit (One Lambda, West Hills, CA, USA), which consists of beads with 1 HLA molecule attached (either class I or class II), referred to as single antigen beads. The procedure was performed according to the manufacturer’s instructions and then analyzed on a LABScan200 flow analyzer (One Lambda). Luminex 100 IS version 2.3 software (Luminex Corporation, Austin, TX, USA) was used for data acquisition, and the data analysis was performed with HLA Fusion software 4.2 (One Lambda). The results were interpreted using MFI values. The samples were considered positive if a Mean Fluorescence Intensity (MFI) value was >1000.

### 2.3. Mononuclear Cell Isolation

Peripheral blood was collected into vacutainer tubes (BD) containing heparin. MNCs were isolated by density gradient centrifugation (Lymphoprep, Axis-Shield, Oslo, Norway), cryopreserved in 10% DMSO (Sigma-Aldrich, St. Louis, MO, USA) and stored in liquid nitrogen until the analysis.

### 2.4. T-Cell Subset Quantification

Fresh whole blood was stained with anti-CD45-FITC, anti-CD3-PC5, anti-CD4-RD1, anti-CD8-ECD (CYTO-STAT tetraCHROME), anti-CD45-FITC, anti-CD3-PC5, anti-CD56-RD1, and anti-CD19 ECD monoclonal antibodies (CYTO-STAT tetraCHROME; all from Beckman Coulter, Milan, Italy). After the lysis of the red blood cells, the absolute CD3 + (Tcells), CD3 + CD4 + (Helper T cells), CD3 + CD8 + (Suppressor T cells), CD3-CD56 + (NK cells) and CD19 + (B cells) (cells/μL) were determined by flow cytometry (Navios, Beckman Coulter) using Flow-Count Fluorospheres in a single platform and lysed no-wash preparation. The gating strategy was set up on CD45 + and side scatter (SSC).

### 2.5. Tregs and Th17 Cells

The MNCs, cryopreserved until the analyses, were thawed at 37 °C and counted for their viability with trypan blue. The cells were suspended at a density of 1 × 10^6^ cells/mL in RPMI supplemented with 10% heat-inactivated fetal calf serum (Euroclone, Milan, Italy) for the cell-surface and intracellular staining, following the standard procedures.

For the Tregs evaluation, after an incubation at +4 °C for 30 min with antibodies specific to cell surface antigens CD4, CD127 and CD25 (Beckman Coulter, Milan, Italy), the cells were treated with fixation/permeabilization buffer (eBioscience, Waltham, MA, USA) at +4 °C for 40 min. The MNCs were washed three times with permeabilization buffer in order to allow intracellular staining for the forkhead box P3 transcription factor (FoxP3) (eBioscience, ThermoFisher Scientific, Waltham, MA, USA) at +4 °C for 30 min.

In order to detect the Th17, the MNCs were stimulated with phorbol 12-myristate 13-acetate (PMA) and ionomycin for 4 h at 37 °C in the presence of brefeldin A. After one rinse, the cells were incubated for 30 min at +4 °C with anti-CD3 and anti-CD4 antibodies (Beckman Coulter, Brea, CA, USA).

After the permeabilization procedure to allow the intracellular staining (eBioscience), the MNCs were incubated with anti-IL17 antibody (Becton Dickinson, Milan, Italy) at +4 °C for 30 min.

For both staining procedures, appropriate isotype-matched controls were used. The acquisition and analysis of the cell populations were performed by direct immunofluorescence using a Navios flow cytometer (Beckman Coulter Life Sciences, Milano, Italy).

Tregs were defined as CD4 + CD127 − CD25 + cells expressing FoxP3. Th17 was defined as CD4 + cells expressing intracellular IL-17.

### 2.6. Assessment of the Graft Function and Proteinuria

Plasma and urine samples were collected at 1, 2, 3 years from the start of the ECP in the R patients, and at 1 and 2 years in the NR patients.

The estimated glomerular filtration rate was calculated using the Chronic Kidney Disease Epidemiology Collaboration (CKD EPI) formula (expressed in milliliters per minute adjusted for body surface area). The creatinine and proteinuria were measured by spectrophotometry (ADVIA xpt, Siemens Healthcare s.r.l, Milan, Italy). We collected eGFR at one year before the start of the ECP from the medical records.

### 2.7. IL 6 Serum Levels

The IL 6 serum level was measured by ELISA using a Duoset kit (R&D system, Space Import Export Srl, Milan, Italy) at the baseline and at one year from the start of the ECP treatment. Briefly, 96-well plates were coated with anti-human IL 6 at 1 µg/mL in a carbonate/bicarbonate buffer pH 9 overnight at room temperature. The plate was washed three times and then treated with 200 µL 2% BSA in PBS for 1 h at room temperature. The serum samples and the standard curve were plated and incubated for 1 h at room temperature. Biotin-conjugated secondary antibody was added for 1 h at room temperature. The plate was washed three times, peroxidase-conjugated streptavidin was added and the plate was incubated for 30 min at room temperature. The plate was washed again three times and tetramethylbenzidine solution (TMB) was added in the dark for 30 min at room temperature; the colorimetric reaction was stopped by adding H_2_SO_4_ 0.18 M. The absorbance was measured at 450 nm (Sunrise—ICN Morisville USA) and the cytokine concentration (expressed in pg/mL) was calculated from the standard curve.

### 2.8. ECP Procedures

The ECP procedures were performed by the local Apheresis Unit. Before each procedure, a complete and differential blood count was obtained. The ECP was performed using the off-line technique, as previously described [40].

Briefly, MNCs were collected from the patient using a last-generation cell separator device, processing 1.5 blood volumes. In order to prevent acide citrate dextrose-related hypocalcemia, calcium gluconate was administered intravenously during the procedure at a mean dosage of 3000 mg, which was increased when symptoms of hypocalcemia appeared (mostly chills and paraesthesias). After the collection, the cells were immediately irradiated (UV-A at 2 J/cmq; Macogenic, Macopharma, France) in the presence of 8-methoxypsoralen (at a concentration of 200 ng/mL). Finally, the photoactivated MNCs were reinfused into the patient. The patient’s vital signs (blood pressure, heart rate, and oxygen saturation) were tested at the beginning and the end of the procedure.

A sample (2 mL) from the leukapheresis collection bag was always obtained for the differential and total blood count in order to detect the MNCs’ purity. The quality controls for the bacteria and fungi detection and UV-A irradiation efficacy were performed as previously described [40].

Arteriovenous fistula (if present) or the radial/ulnar vein were used as the venous access.

The ECP treatment schedule was borrowed from the protocol used in our Centre for lung chronic rejection, and was modified as follows: 1 cycle (i.e., 2 procedures) per week for 3 weeks, 1 cycle fortnightly 2–3 times, 1 cycle per month if the patient was improved/stabilized; then, the patients were maintained chronically on ECP, progressively lengthening the treatment intervals to 2 months (Supplementary Figure S1). The ECP was temporarily suspended in the case of an onset of fever or intercurrent infection, or an absolute MNCs count in the peripheral blood of <200 × 10^9^/L.

### 2.9. Statistical Methods

The continuous data were described with the mean and standard deviation (SD) or the median and quartiles (IQR), depending on the distribution and categorical data with the counts and percentage. Given the exploratory nature of the study, no *p*-values are reported.

GraphPad Prism version 8.2.1 (GraphPad Software, San Diego, CA, USA) and Stata 16 (Colleg Station, TX, USA) were used the computations.

## 3. Results

### 3.1. Baseline Patient Characteristics

All of the patients but one were transplanted in our Centre from 2008 to 2014. The demographic and clinical characteristics are reported in Table 1. The average age was 49.5 ± 8.87 years (M ± SD) and the sex distribution was similar. All of the patients were Caucasian, except for one African. Thirteen grafts were retrieved from cadaver donors and one from a living donor. In all of the patients, the Panel Reactive Antibody (PRA) was negative at the time of grafting, except for one that was 30%. Three episodes of acute rejection and one delayed graft function occurred in three different patients. The HLA match ranged from 2 to 5. The median time from transplant to enrolment was 9.25 years (IQR 6.66–11.9 years). The immunosuppression therapy included basiliximab, calcineurin inhibitors, mycophenolate mofetil, steroids and mTOR inhibitors in various combinations tailored to the individual patient’s needs. The mTOR inhibitors were replaced by calcineurin inhibitors before enrolment (Table 1).

Class II DSA were found in eleven patients and class I DSA was found in one patient. All of the DSA were *de novo*. In one patient, class I and II anti-HLA antibodies were found, but the donor specificity was ignored. At the enrolment in the photopheresis treatment, the eGFR median was 31.65 mL/min (IQR 24.83–44.98 mL/min) and the 24-h proteinuria median was 1.2 g (IQR 24-h proteinuria 0.46–2.35 g). All of the patients were treated with ACE inhibitors or Angiotensin II receptor blockers.

Among the 14 enrolled patients, three stopped the ECP within 6 months for logistical and/or personal reasons, and one patient stopped after 24 months for his will.

The median number of ECP sessions performed by each patient was 46 (range 11–87), while the median number of photoactivated and reinfused MNCs was 0.91 × 10^9^ cells/kg (IQR 0.63–2.49 × 10^9^ cells/kg) for each procedure. No complications occurred during the ECP and no relevant alteration in the hemodynamic parameters was observed; furthermore, no hemorrhage or local bleeding occurred in the patients under anticoagulant therapy. No MNC reinfusion-related adverse events were observed. There was no red blood cell transfusion or albumin infusion requirement. We recorded hemorrhagic cystitis and cutaneous Herpes Zooster viral infection reactivation in one patient after 5 and 9 months from the start of the ECP, respectively, and a cutaneous Herpes Zooster viral infection reactivation after 4 months from the start of the ECP in another patient.

### 3.2. Effects of ECP Treatment on the Estimated Glomerular Filtration Rate and Proteinuria

**Figure 1:**
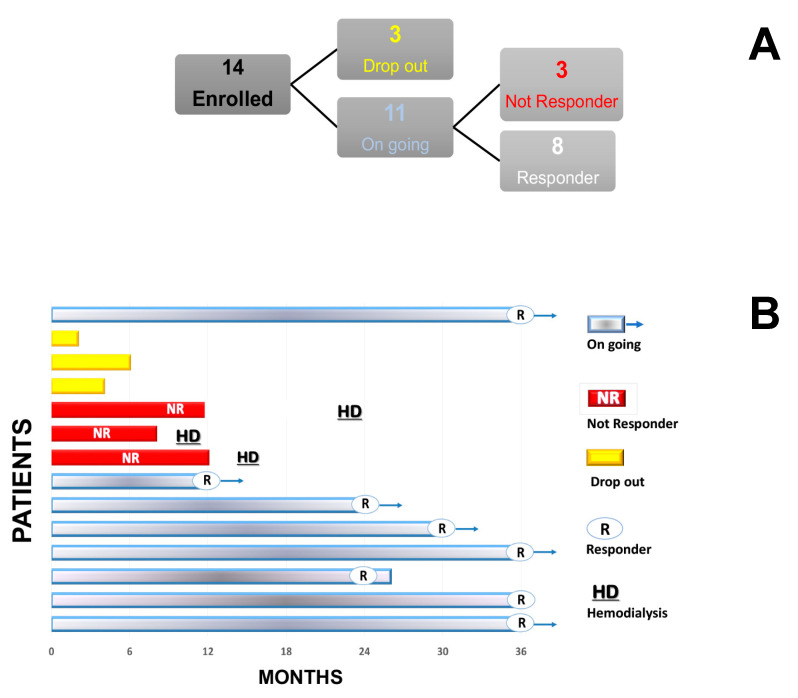
Panel (**A**), flow chart: 14 patients were enrolled, three stopped ECP treatment within 6 months for logistical and/or personal reasons, and one patient stopper after 24 months for his will. Panel (**B**): the clinical outcome of the enrolled patients. R, responder patient; NR, non-responder patient; HD, haemodialysis; Ongoing, patients that continued ECP treatment; Drop out, patients that stopped the ECP treatment.

We excluded from the analysis three patients due to early and voluntary ECP treatment withdrawal (Figure 1A).

**Figure 2:**
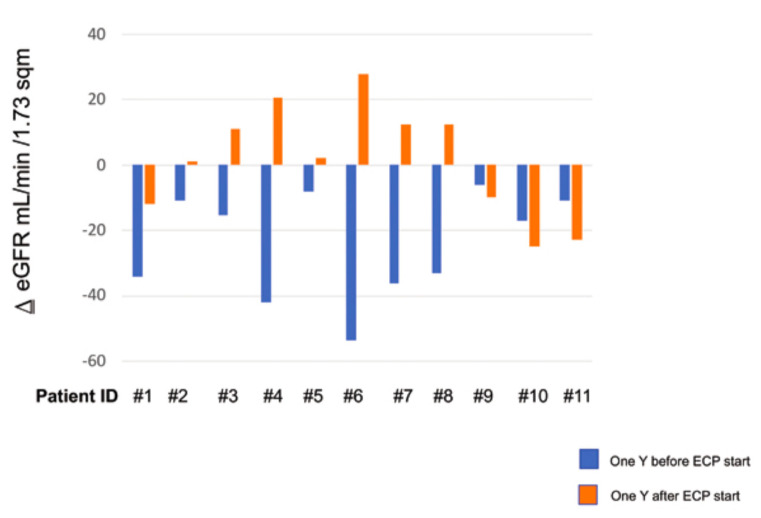
Estimated glomerular filtration rate (eGFR) was evaluated 1 year before and after the ECP treatment in all of the patients, except for the three drop out patients. The blue columns represent the eGFR before the ECP treatment. The orange columns represent the eGFR after the ECP treatment. The data are expressed as Δ eGFR mL/min/1.73 m^2^.

**Figure 3:**
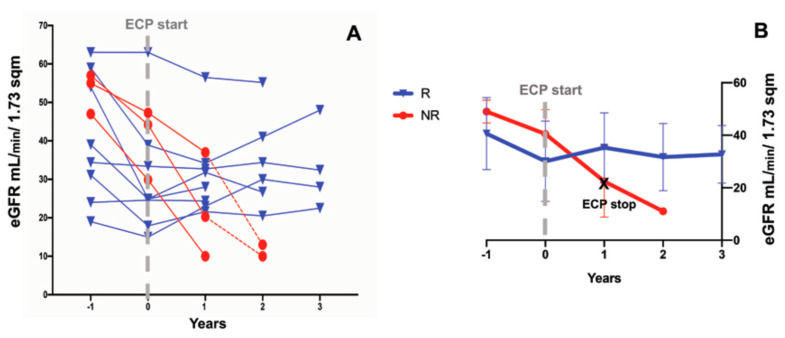
Renal function pre- and post-ECP treatment. Panel (**A**): The estimated glomerular filtration rate (eGFR was evaluated 1 year before and after the ECP treatment in responder (R) patients (blue line) and the non-responder (NR) patients (red line). In the R patients, the ECP treatment was prolonged by up to 3 years, showing the stabilization of the eGFR at the last follow up. In the NR patients, the follow up was prolonged after the discontinuation of the ECP for up to 1 year in two patients (spaghetti plot). The red dashed line marks the follow up after the ECP’s stoppage in the NR group. The dashed gray line indicates the start of the ECP treatment. Panel (**B**): The eGFR trend over time in the R (blue line) and NR (red line) patients. The dashed gray line indicates the start of the ECP. The black cross marks the end of the ECP treatment in the NR group. The data are expressed as the mean and SD.

One year before the start of the ECP start, the eGFR rate decline was similar in all of the patients. One year after the start of the ECP, the primary endpoint (i.e., eGFR not decreasing by more than 20% with respect to the baseline) was attained in eight (72.7%) patients; in addition, in seven of them (63.6%), the eGFR rose rather than decreasing. Only in three out of 11 patients (27.2%) did the eGFR worsen, declining by more than 20% (NR) (Figure 2. Figure 3A). In the last patients, the ECP treatment was stopped according to protocol, and hemodialysis was started 4–12 months after the withdrawal of the ECP (Figure 1B). At the baseline (T0), the average eGFR was similar in both groups. Because no adverse event was recorded in the R patients, the ECP treatment was prolonged and we observed a stabilization of the eGFR over a 36-month follow-up (Figure 3B).

**Figure 4:**
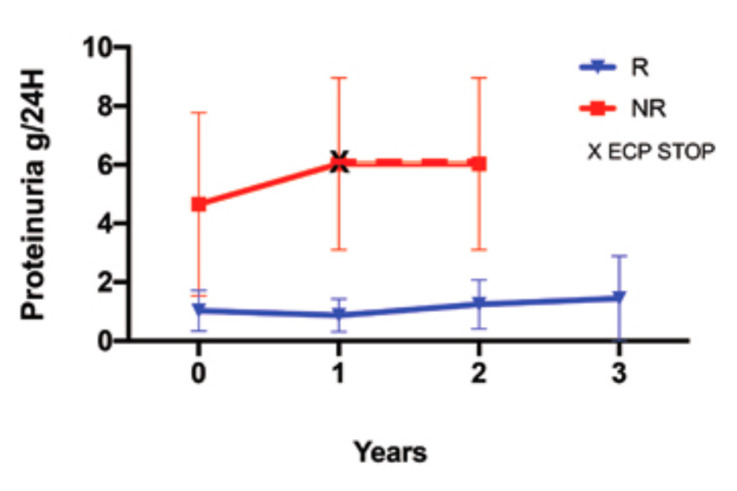
24 h proteinuria was evaluated after the ECP treatment in the responder (R) patients (blue line) and the non-responder (NR) patients (red line). In the R patients, the ECP treatment was prolonged by up to 3 years, showing the stabilization of the proteinuria at the last follow up. The black cross marks the end of the ECP treatment in the NR group. The red dashed line marks the follow up after the ECP stoppage in the NR group. The data are expressed as the mean and SD.

Twenty four hour proteinuria did not increase in the R patients throughout the follow up, as opposed to the NR. It is noteworthy that the 24-h basal proteinuria of the NR group was higher than that of the R group (M ± SD, NR: 24-h proteinuria 65 ± 3.11 g; R: 24-h proteinuria 1.02 ± 0.69 g) (Figure 4).

The number of photoactivated MNCs reinfused in each procedure did not differ between the R and NR patients (R median: 0.76 × 10^9^ cells/kg IQR 0.59–2.0 × 10^9^ cells/kg; NR median: 0.91 × 10^9^ cells/kg IQR 0.76–2.49 × 10^9^ cells/kg).

### 3.3. Anti-HLA Antibodies

In the R patients, we observed a decrease of the anti-HLA antibody serum levels after the ECP treatment. In particular, we found a complete antibody clearance in six (75% of R) patients; in five of them, after 1 year of ECP onset; and in one after 2 years. In all of the NR patients, the anti-HLA antibody (DSA and not DSA) serum levels did not decrease after the onset of the ECP; additionally, we observed an increase of the anti-HLA antibodies (DSA and not DSA) after the discontinuation of the ECP (Table 2 and Supplementary Figure S2).

### 3.4. Immune Cell Subpopulations

**Figure 5:**
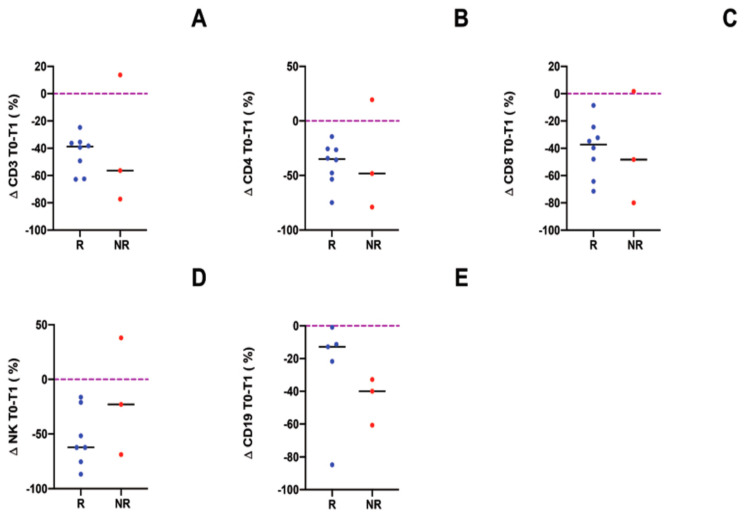
Immune cell subpopulations: CD3 (**A**), CD4 (**B**), CD8 (**C**), NK (**D**), CD19 (**E**) cells were analyzed in all of the patients, except in one R patient and one dropout patient. The data are expressed as Δ percentage of the cells’ number before (T0) and after 1 year (T1) of ECP treatment.

The immune cell subpopulations were studied in all but one patient. The observed patients showed a decrease of CD3, CD4, CD8, NK and CD19 cells after one year of the ECP treatment (the data are expressed as ∆ percentage) (Figure 5A–E).

**Figure 6:**
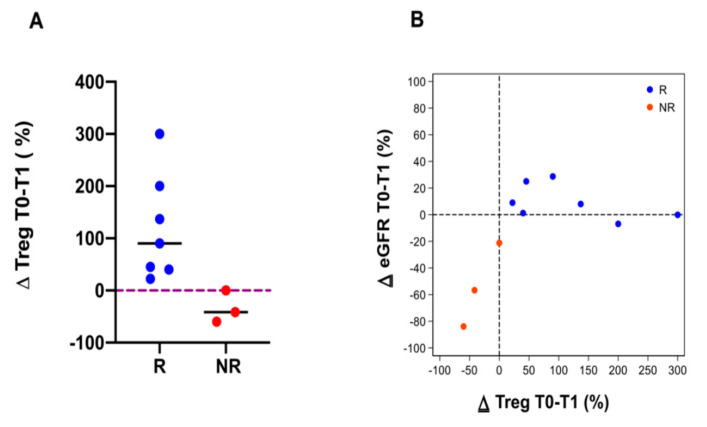
Immune cell subpopulations (CD3, CD4, CD8, NK, CD19 cells) were analyzed in all of the patients, except in one R patient and the dropout patients. The data are expressed as Δ percentage of the cells’ number before (T0) and after 1 year (T1) of ECP treatment (**A**,**B**).

In the R group, the ECP treatment was associated with a Tregs cell increase. In the NR group, the Tregs decreased in two patients and did not change in one patient after the start of the ECP (Figure 6A). In addition, we found a direct correlation between ∆ GFR and ∆ Tregs in the NR group (Figure 6B).

**Figure 7:**
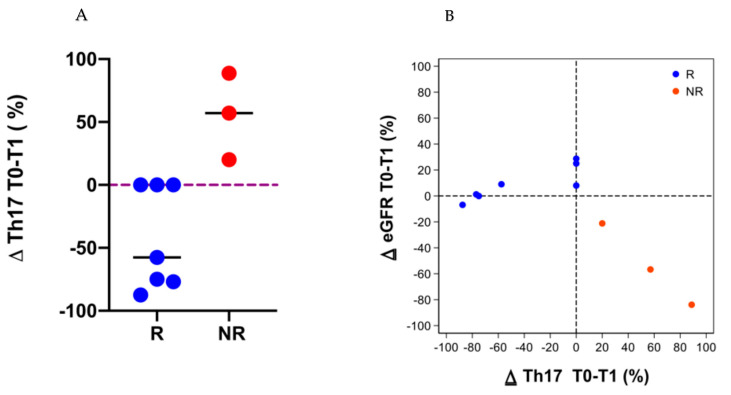
Th17 cells were analyzed in all of the patients except in one responder (R) patient and the dropout patients. Panel (**A**): The data are expressed as Δ percentage of the Th17 cells’ number before (T0) and after 1 year (T1) of ECP treatment. The blue dots represent R patients and the red dots represent the non-responder (NR) patients; the line represents the median. Panel (**B**): A scatter plot illustrating the relationship between the Δ percentage of the Th17 cells and Δ eGFR in the R and NR patients. The blue dots and red dots represent R and NR patients, respectively. The diagram shows an inverse correlation between the Δ percentage of Th17 cells and the Δ eGFR in NR patients.

Inversely, in the NR group the Th17 cells increased after 1 year of ECP treatment, while in the R group the Th17 cells decreased in four patients and did not change in three patients (Figure 7A). Furthermore, we found an inverse correlation between ∆ GFR and ∆ Th17 in the NR group (Figure 7B).

### 3.5. Serum IL 6 Levels

**Figure 8:**
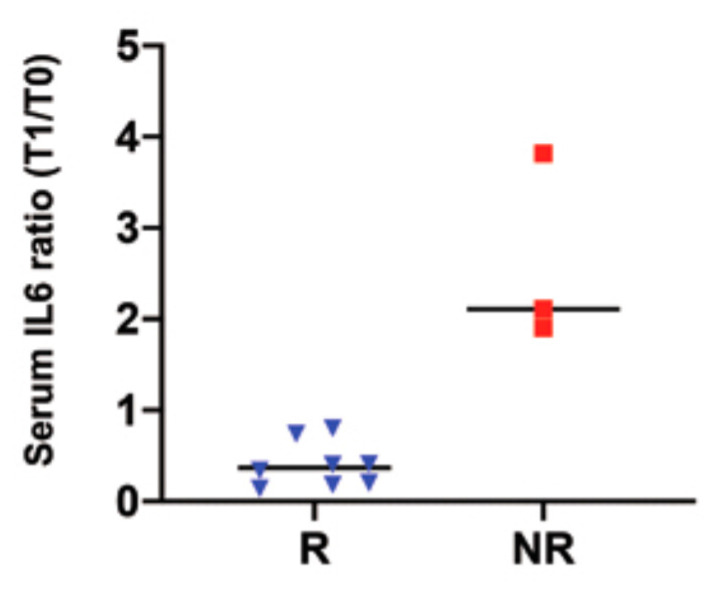
IL 6 ratio between the IL6 serum levels after 1 year of ECP treatment (T1)/baseline levels (T0) in the R and NR patients. The blue dots and red dots represent the responder (R) and non-responder (NR) patients, respectively.

In the R patients, we observed a reduction of the IL 6 serum levels after one year of ECP, unlike for the NR patients. The ratio of serum IL 6 after ECP/before ECP was lower in the R than in the NR group (Figure 8).

## 4. Discussion

In this study, we meant to address the present need for new therapeutic strategies against the chronic rejection of kidney transplants, a disorder that ranks first as the cause of graft loss and is unresponsive to the available therapies. We were induced to explore the effects of ECP by the recent evidence of its effectiveness in chronic lung rejection [37].

The preliminary results of our prospective observational investigation confirmed that ECP is a concrete effective weapon to combat chronic ABMR, providing a sound base for further confirmatory investigations. Until now, ECP has been used in renal transplants in a very limited number of patients with acute rejection, and in anecdotal cases of chronic rejection. In the largest study, ECP was delivered to 33 patients with circulating DSA, but only two had chronic rejection; the remaining 31 had acute rejection, and in eight out of them the rejection was cell mediated. ECP stabilized the renal function in one third of the patients, including the two cases of chronic rejection [36]. Gathering results from the case reports or collections of few patients, about 50% of patients with acute rejection benefitted from ECP, while the anecdotal cases of chronic kidney rejection showed variable results. The analysis of these few cases suggests that high serum creatinine levels, severe histopathological scores and a delay in delivering ECP select non-responder patients.

Different timings and numbers of sessions were additional confounding factors [29,30,32,35,41,42,43].

Our protocol aimed to evaluate the effects of 1-year ECP treatment on renal function, but in the R patients, we reported data on the effect of ECPs beyond the year; because the ECP treatment showed a beneficial effect and no adverse complications, it was prolonged up to 36 months.

ECP blocked the descent of GFR in eight out of 11 patients (not 14, because three patients dropped out for personal reasons), and in seven of them eGFR even increased, a therapeutic impact that went beyond our best expectations. It is interesting to note that in the NR patients with baseline eGFR and histological scored similar to the R patients, the proteinuria was a lot higher. However, the number of patients is too low to consider the proteinuria value at the baseline as predictive factor.

A major finding of our study was the significant lowering of the DSA levels after the ECP treatment. The DSA decreased gradually and disappeared in 75% patients. Similar results were shown by Gautam Baskaran et al. in patients with lung transplants and bronchiolitis obliterans, but unlike our patients they had previously been treated with ATG and/or rituximab [44].

Several clinical and experimental studies have demonstrated that lesions induced by DSA in the endothelial cells of peritubular and glomerular capillaries gradually evolve to an irreversible stage and permanently compromise the graft function [6].

Moreover, the decline in the DSA levels is associated with better graft survival. De novo DSA but not pre-existing DSA predict a worse graft outcome in patients with chronic ABMR [45,46,47].

Our results confirm that the abatement of anti-HLA antibodies improves the graft outcome and indicates that the time necessary for DSA depletion to affect the clinical outcome has to be longer than one year. We emphasize this point because, so far, no one has ever prolonged the ECP treatment for so long; instead, the ECP duration has an important impact on the results.

The possible mechanisms underlying the actions of ECP have not been exhaustively explored, but they include the induction of T cell apoptosis by UVA irradiation in the presence of 8-MOP, the ensuing shift of T helper cells to the Th2 subset, the downregulation of pro-inflammatory cytokines, enhanced anti-inflammatory responses and the upregulation of Tregs cells [29,30,35,42,43,48].

We have addressed the point by analyzing the immune cell profile, and we found in all of the patients a decrease of the CD3, CD4, CD8, CD19 and NK cells. The reduction of the NK cells is noteworthy, as NK cells have a central role in the pathophysiology of chronic ABMR [49]. Many studies have already demonstrated the central role of Tregs cell in inducing immune tolerance and the ability of Tregs to downregulate the response of CD8, CD4 and NK cells. Additionally, there is evidence that, in allograft models, the antagonism of the IL-17 network can reduce the intra-graft production of inflammatory cytokines and prolong graft survival. Moreover, the respective roles of T cell subtypes seems to depend on the cytokine environment induced; in fact, it has been shown that IL 6 deviates the T helper cells to a prevalent Th17 phenotype [49]. Our findings show a significant decrease of IL 6, paralleled by an increase of Tregs and a decrease of Th17 cells after ECP treatment in the R patients. No-one has, thus far, explored the effect of ECP on Th17 cells; here, we add a piece to the ECP complex mechanism of action, i.e., the ECP-induced downregulation of Th17 cells.

Finally, it is important to emphasize that ECP did not cause adverse events and infective complications. The treatment schedule was the same as that used in our hospital for patients with chronic lung rejection, although we do not know whether a different schedule or customized ECP treatment could lead to better results. The technique was “off-line” because it offers the advantage of a low extracorporeal volume and a short procedure time (about 120 min). This last advantage should not be underestimated because, as such, it is acceptable to patients that are emotionally sensible to any procedure reminding them of their past hemodialysis experience. Moreover, the off-line technique gives us the possibility to perform quality controls on the product collected and reinfused after the UV-A irradiation [40].

In summary, we have given preliminary evidence that ECP treatment abates the anti-DSA antibody titer and blocks the progression of chronic ABMR, as demonstrated by the stability of the eGFR throughout the follow up. Our results support the immunoregulatory effect of ECP on the cellular and humoral responses. This is the first successful treatment of chronic ABMR obtained in the absence of side effects, and it is the first that evaluates the effects of such a long-term ECP therapy. In spite of the methodological limits of a prospective observational study, our results provide a sound base for the further testing of ECP in a multicenter randomized study.

## Figures and Tables

**Table 1:** Demographic and clinical characteristics of the enrolled patients at the baseline.

Variables	*n* = 14 Patients
Sex	
Male (%)	42.8
Female (%)	57.2
Age (years)	
Mean ± SD	49.55 ± 8.87
Ethnicity	
Caucasian (%)	13 (92.86)
African (%)	1 (7.14)
Cause of ERSD	
Unknown (%)	3 (21.43)
GN (%)	4 (28.57)
ADPKD (%)	1 (7.14)
HT (%)	1 (7.14)
DN (%)	0 (0)
Other genetic (%)	2 (14.29)
Other (%)	3 (21.43)
Pre-transplant dialysis duration (years)	
Mean ± SD	3.9 ± 3.0
Donor Type	
Deceased (%)	13 (92.86)
Living (%)	1 (7.14)
Retransplantation	
Yes (%)	1 (7.14)
No (%)	13 (92.86)
PRA (%)	
Median; min-max	0 (0–30)
HLA match	
Median; min-max	2 (2–5)
DGF	
Yes (%)	1 (7.14)
No (%)	12 (85.71)
Unknown (%)	1 (7.14)
Acute renal allograft rejection	
Yes (%)	3 (21.4)
No (%)	9 (78.6)
Transplant age (years)	
Median and IQR	9.25 (6.66–11.9)
Immunosuppressive therapy (%)	
Induction	9 (64.29)
Basiliximab	5 (35.71)
Anti Thymocyte globulin	
Maintenance	6 (42.86)
Cyclosporin	8 (57.14)
Tacrolimus	1 (7.14)
mTOR inhibitor	12 (85.71)
Corticosteroids	14 (100)
Mycophenolate mofetil/Mycophenolic acid/Azathioprine	

SD = standard deviation, ESRD = End Stage Renal Disease, GN = Glomerulonephritis, ADPKD = Autosomal Dominant Polycystic Kidney Disease, HT = Hypertension, DN = Diabetic Nephropathy, PRA = Panel Reactive Antibody, DGF = Delayed Graft Function, IQR = Interquartile Range.

**Table 2:** Anti-HLA antibody (DSA and not DSA) serum levels expressed as the mean fluorescence intensity (MFI) at the baseline and after the ECP treatment.

ID Patient	DSA	Anti-HLA-Ab	DSA (MFI) Baseline	DSA (MFI) 1Y	DSA (MFI) 2Y	DSA (MFI) 3Y
# 1	DQ7		36,780	31,500	18,330	26,000
	DQAI		21,811	18,800	13,750	18,000
# 2	DQ4		12,000	4890	Neg	Neg
	DQ4		6800	3800	Neg	Neg
	DQ6		3900	1700	Neg	Neg
# 3		CW7	3000	Neg	Neg	Neg
# 4	B47		2197	Neg	Neg	
# 5	DQ5		5474	5000	Neg	
	DQA		3045	Neg	Neg	
# 6	DQ61		15,800	Neg	Neg	
	DQ62		4000	Neg	Neg	
	DQ53		19,120	Neg	Neg	
	DQ64		3785	Neg	Neg	
	DQ69		14,000	Neg	Neg	
# 7	DQ7		47,000	27,000		
# 8	DR11		4000	Neg	Neg	Neg
	DR15		1500	Neg	Neg	Neg
ID Patient	DSA	Anti-HLA-Ab	DSA (MFI) Baseline	DSA (MFI) 1Y	DSA (MFI) 2Y	DSA (MFI) 3Y
# 9	DQ21		17,700	17,000	15,562	19,623
	DQ22		5000	1900	4902	6170
	DR531		5500	2000	2168	1962
	DR533		5300	4300		
# 10		see Supplementary Figure S2				
# 11	DQ21		4215	17,660		
	DQ22		18,324	37,689		
	DR531		6715	11,217		
	DR533		0	2062		

DSA = Donor Specific Antibody, Ab = Antibody, MFI = Mean Fluorescence Intensity, Y = Year.

## Data Availability

Not applicable.

## Supplementary Materials

The following are available online at https://www.mdpi.com/article/10.3390/biology10060547/s1, Figure S1. Schedule of ECP treatment. 1 cycle of ECP included two sessions performed in two consecutive days. Figure S2. Anti HLA antibodies serum levels of not responder patient, whose HLA typing was unknown.

## Abbreviations

ABMR: antibody-mediated rejection; CKD EPI: Chronic Kidney Disease Epidemiology Collaboration; CTN: chronic transplant nephropathy; DSA: donor-specific alloantibodies; ECP: extracorporeal photopheresis; FoxP3: forkhead box P3 transcription factor; GVHD: graft versus host disease; HLA: human leukocyte antigen; IL-6: interleukin 6; IVIg: intravenous immunoglobulin; MFI: mean fluorescence intensity; MNCs: mononuclear cells; NR: non-responder patients; PRA: panel reactive antibody; PTU: proteinuria; R: responder patients; SD: standard deviation; Th17: T helper 17 cells.
