# Supplementary material for: Photopheresis Abates the Anti-HLA Antibody Titer and Renal Failure Progression in Chronic Antibody-Mediated Rejection
Source: Biology (Basel). 2021 Jun 18;10(6):547. doi: 10.3390/biology10060547 (PMC8234140; doi:10.3390/biology10060547)
Supplement: Supplementary file 1 [file biology-10-00547-s001.zip › biology-1240095-supplementary.pdf]

biology

MDPI

Supplementary

Photopheresis Abates the Anti-HLA Antibody Titer and Renal Failure Progression in Chronic Antibody-Mediated Rejection

Marilena Gregorini, Claudia Del Fante, Eleonora Francesca Pattonieri, Maria Antonietta Avanzini, Maria Antonietta Grignano, Irene Cassaniti, Fausto Baldanti, Giuditta Comolli, Angela Nocco, Miriam Ramondetta, Gianluca Viarengo, Vincenzo Sepe, Carmelo Libetta, Catherine Klersy, Cesare Perotti and Teresa Rampino

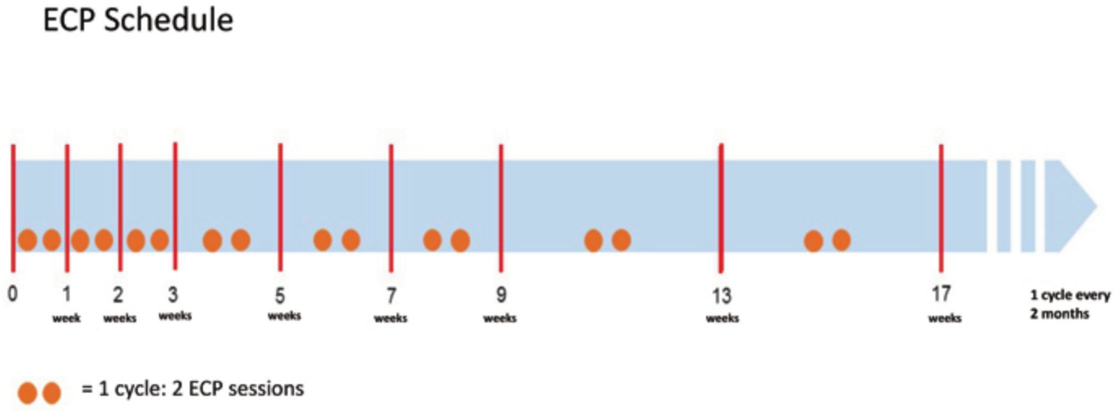

Figure S1. Schedule of ECP treatment. 1 cycle of ECP included two sessions performed in two consecutive days.

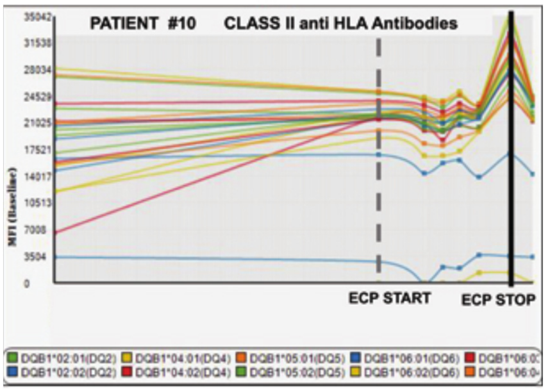

Figure S2. Anti HLA antibodies serum levels of not responder patient, whose HLA typing was unknown.
